# Address to the Graduating Class of the Balto. College Dental Surgery

**Published:** 1881-03

**Authors:** William Kirkus


### ARTICLE III.

### *Address to the Graduating Class of the Baltimore College of Dental Surgery.*

DELIVERED BY REV. WILLIAM KIRKUS, M. A., LL. B.

It would have seemed far better to me, gentlemen, if the duty that has been assigned to me had been entrusted to some one of the noblest and wisest of the members of your own profession. The wisdom of such a man, his experience, his success, his wide-spread renown, would have given weight to every word he would have uttered. He could have warned you, from his own knowledge, of the temptations which will beset you in the career that you have chosen. He could have shown you the traps and pitfalls which undoubtedly are hidden in the road on which you are to journey. He could have given you suggestions which would have helped you to tread boldly and safely, and to march on surely and perhaps even swiftly, to a dignified success. It is impossible for me to render you any such service. The life you will have to live is, in many respects, very remote from that with which I am myself familiar. I know nothing whatever about teeth, except that (fortunately for you) they must generally be numbered among the "ills that flesh is heir to;" nor can I render you the slightest assistance, even by way of suggestion, in your philanthropic endeavor to fortify, that *erkos odonton* about which you used to read in Homer. Nevertheless it is not for me to dispute the wisdom of your professors—I can only wish that the task which they have assigned to me had been entrusted to far abler hands. Perhaps it may be sometimes an advantage to receive honest and generous advice, or even criticism, from one who looks at us "without partiality and without hypocrisy;" one who does not belong to our own set; one who does not judge by our own standard; one who knows that there are other things worth living for besides those which are the objects of our own too exclusive pursuits. You are but men after all, even though you be dentists. There are even some physicians, as there are men in all professions, not excluding the clergy, to whom might be addressed without inappropriateness, the words of the immortal Venus in "Our Mutual Friend"—"you little know how small you would look if I had the articu lating of you." Or to put it more generously, and in words yet more familiar:—

> O wad some Pow'r the giftie gie us
> To see oursels as others see us!
> It wad frae mony a blunder free us
> And foolish notion:
> What airs in dress and gait wad lea'e us,
> And ev'n Devotion!

Or yet again, you may be so accustomed to your own work that you may forget its real dignity, underestimate its power, and so be in danger of missing that highest success which can only be achieved by men who believe in themselves and have an enthusiasm for their vocation.

I mentioned as one among many causes which may render me, I fear, a very inefficient counsellor to young physicians, that the life on which you are setting out must be in very many of its details exceedingly unlike my own. And yet on the other hand there are many points of strong resemblance; and perhaps the resemblance, being deep and general, may be more important than the differences which are superficial and in details. To begin with, then, we both belong to what are called the professional classes. It would be absurd, even to the verge of insanity, to underestimate or disparage the tradesman class. Not only does this include some of the very best men on the face of the earth, but their work itself—to say nothing of its absolute necessity—may be made, and often is made, in the very highest degree ennobling. Moreover trades are not separated from professions by any "hard and fast" line. When a hairdresser calls himself Professor Snippers, you inevitably set him down as a fool; though very likely he is a very excellent fellow and his wife never saw his equal. Nobody would call oyster packing a profession, nor speak of Crosse & Blackwell as Professors of Pickling. Unless indeed, with our modern notions of liberty, we felt free to invert the meaning of perfectly familiar words—or used the term professor with a covert irony—as we should do if we were to call Judas Iscariot a professor of devoted loyalty, or compliment an amœbus on its altruism. But there are large commercial transactions which require such vast and minute knowledge, such versatility, such large powers of generalization and deduction, such familiarity with the laws and usages of many countries and all classes of society, and in the long run, such sterling honour—that those who conduct them are justly ranked among the wisest and most cultivated of mankind. A banker may be, and possibly often is, a mere trader; but he may also be, and ought to be, familiar at least with the methods and results of political economy, with the theory of currency and exchange, and the innumerable forms and effects of credit.

But speaking broadly and without any pretence of a strictly accurate definition, the difference between a trade and a profession is this: that a trade is a mere instrument, the means to some end other than itself, whereas a profession is an end in itself. A tradesman is not generally—always taking for granted, of course, honesty, and average skill—responsible for what he sells. *Caveat emptor*—let the buyer look to it, is the motto that might be written up in large letters in every store. Moreover a storekeeper will sell you anything you want to buy. He will sell you a good article, if you will pay the price. But if you choose he will sell you a really bad article, which both you and and he know to be bad. It is your own look out. His object—and a very legitimate object—is to make money:

and therefore he must accommodate himself to the taste of his customers, however ridiculous or even mischievous those tastes may be. An honest jeweller will of course tell you whether his gold is pure and his precious stones genuine. But if you are so hopelessly mad that you would rather wear gilded nickel and coloured glass on your fingers than be suspected of having no jewelry at all—of course he will sell you that. But no physician could act in that way. His end is not money but his profession, his work, the cure of his patient. If he thinks some particular medicine necessary he will prescribe it; and if you are so ridiculous as to insist upon something else he will politely bid you good bye. Remember always, gentlemen, that you belong to a profession not to a trade-guild. If you do remember that, you will be both modest and dignified; you will be protected not only from the whims and caprices of others, but from the far more serious dangers which you will be not long in detecting concealed in your own hearts. What would you think of a Christian priest who, instead of delivering what he believed to be God's message to the souls of men, should first discover their own preferences and then, in the name of the Eternal, echo to them their own voices? And next in ideal nobleness to the work of him who brings divine messages to the spirits of men, is the work of him who brings the divine gift of healing to their bodies. If you should condescend to pander to popular prejudice, if you should sacrifice what is really good for popular applause, if you should be satisfied not with what is best but with what 'will do,' should you make it the end of your work not to bring health and healing to your patient but to extract fees out of them—then whatever your prosperity, whatever your reputation, whatever your wealth, you will have sunk into an abyss of unmeasurable infamy. I do not say, hate quacks as you would hate the devil—for indeed, apart from better reasons, you are likely to have enough of that feeling already—but I will say, hate quackery as you would hate hell.

Again—and perhaps this should have come first, for it is indispensable to your genuine success—never forget that you are *gentlemen*. And by this I do not mean that you are well-bred, that you came of a good stock, that you have hanging up in your library or office a stately genealogical tree. No doubt this would be a very great advantage. It is a splendid introduction; it gives a man courage, brings out his personal nobleness, and is a powerful stimulus to exertion. I have observed that the most democratic of politicians are often among the most aristocratic in society. It is a good thing to have a father to be proud of. But nevertheless, any defects or misfortunes of your birth it would now be too late to remedy. We may do much in a free country, but not even the Constitution of the United States can enable us to abolish the Multiplication Table or to change our fathers and mothers.

But by a gentleman—such as any of us may become—I mean an educated man, a man familiar with the approved usages of good society, a man who knows how to walk without treading on other people's feet, who knows how to talk without outraging other people's feelings,—a good-hearted, unselfish, modest man—a man not only ready to do a kindness, but also humble enough and generous enough to accept one—a man who "envieth not, vaunteth not himself, is not puffed up, doth not behave himself unseemly, is not easily provoked, thinketh no evil, rejoiceth not in iniquity but rejoiceth in the truth"—a pure man, with a clean soul and an undefiled life. Do not think that knowing all about teeth will relieve you from the necessity of knowing something about good books and great men. I once heard of a candidate for Holy Orders who spelt Peter, P E T R E, and felt himself deeply wronged because the examining chaplain refused to pass him. I once read a letter from an M. D. equally independent of Webster Unabridged. But really people have an invincible prejudice in favor of correct spelling, which you cannot afford to despise. If you spell like Sairey Gamp and Betsey

Prig you must not be surprised if people don't consult you. Why a well-educated girl might even, for so very simple and rudimentary a reason, refuse to marry you. What would Major Pendennis say to such a match? You remember his splendid strategy in rescuing his nephew from the too attractive Miss Costigan. "I *will* see her," said Arthur, "I'll ask her to marry me once more. I will. No one shall prevent me." "What! a woman that spells affection with one f? Nonsense, sir; be a man, and remember that your mother is a lady."

But, indeed, your profession will often place you in circumstances of extreme delicacy—requiring not only the manners but the feelings of a gentleman. We all of us remember the solemn awe with which we first surrendered our very windpipes to the glittering weapon of a hitherto unknown barber. What if he bore us malice for some unknown injury or slight! What if he were an unsuspected maniac! Think then, of the awe and dread of a blushing maiden surrendering her face, her mouth, her very teeth, to the grappling irons, or saws, or files, of a hitherto unknown dentist! Besides if you reside in some country town or village—and somebody must live in such places, and they even come to enjoy them—you will be one of the leaders of society. In the desert of a large city you might "blush unseen," but "down there" everybody will know you. You will be, or ought to be, not only a dentist but a social power. You may set a fashion—not in neckties, and tight-fitting suits and all the matchless perfections which distinguish that marvellous creature—that last and noblest birth of time—who is sometimes irreverently spoken of, in the gentle slang of the day, as a 'lum-tum'—not in all this; but in manners, in good sense, in solid worth, in courtesy, and refinement, and culture. Take care to do this part of your work well—and be in every respect a gentleman.

It may perhaps seem entirely superfluous to remind you that whatever else you are, it will unquestionably be necessary for you to be physicians. Of course, you know perfectly well what this means, but I am sure there are a great many people, and myself among the number, who do not. Of course we all know perfectly well what a physician's education should be ; and if we did not know it otherwise, we might easily find it out by reading the regulations of your own college. Thus, for instance, we should find that your course of study includes Pathology, Therapeutics, Anatomy and Physiology, Chemistry and Materia Medica. Nobody of course suspects that you have confined your study to the structure of the teeth, or the mechanical appliances which are necessary for cleaning, stopping, extracting or imitating them. Whatever your private practice may be, it is to be presumed that you would be able, if you thought fit, to undertake the general medical treatment of any patient who might be willing to entrust himself to your care. But what I mean by uncertainty, as to what a physician really is, is something entirely different from this. What is it that gives a man a right to practice medicine—to undertake the care of one of his fellow-creatures even in a case of life and death, or where the comfort of a whole lifetime may depend upon his knowledge and skill ? Now in England, where as you know, people have a very practical and humdrum way of doing things, people would never be content for a week to be left in any uncertainty on a matter of so much importance. First of all, there are exceedingly few institutions that are authorized to confer degrees in medicine. For England itself there are, as far as I can remember, only three ; the Universities of Oxford, Cambridge and London. Indeed it is the University of London alone, which has devoted a very special attention to all departments of physical science. Anyone who looks down the comparatively short list of London medical graduates, will come across some of the foremost names in the profession ; and I advise you to examine the list for yourselves, lest you should suspect me of partiality for a University of which I am myself proud to be a member. Almost everybody in England knows, that as a matter of mere business, a London medical degree is an almost certain introduction to a lucrative practice. But in England, not even a medical degree—rare as such a distinction is—confers a right to practice medicine; unless the law has been altered within the last eight years. Before a man can practice medicine he must take care to have his name inserted in the authorized register; and before he can secure this, he must obtain the necessary license from one or other of the three great medical corporations—the Royal College of Physicians, or of Surgeons, or the Apothecaries' Society. If without this authority he chooses to call himself a doctor, to visit patients, and to demand from them fees, he can be prosecuted for the very vulgar crime of obtaining money under false pretences. If in the care of a patient —through a surgical operation or a mistake of medical treatment—he should sacrifice limb or life, he would have no other protection than any private individual in defending himself against legal proceedings. An authorized medical practitioner in England, then, is a man whose name appears in a published list drawn up according to the directions and by the authority of an Act of Parliament.

[TO BE CONTINUED.]

